# A study on the influence of rural tourism’s perceived destination restorative qualities on loyalty based on SOR model

**DOI:** 10.3389/fpsyg.2025.1529686

**Published:** 2025-04-07

**Authors:** Ning Zhu, Haochen Xu, Xi Zhang, Li Chen

**Affiliations:** ^1^School of Architectural Art and Design, LuXun Academy of Fine Arts, Shenyang, China; ^2^Shanghai Academy of Fine Arts, Shanghai University, Shanghai, China; ^3^School of Design, Jianghan University, Wuhan, Hubei, China

**Keywords:** perceived destination restorative qualities, hedonic experience, eudaimonic experience, destination image, loyalty, SOR model

## Abstract

**Introduction:**

Tourists’ restorative perception of the environment in rural tourism is a key factor to enhance tourism experience and promote physical and mental recovery, as well as a new perspective of tourist psychological research. However, the current research on the impact of tourists’ perceived destination restorative qualities on loyalty in rural tourism has not been deeply discussed.

**Methods:**

Based on the Stimulus-Organism-Response (SOR) framework, this study explores the relationship between tourists’ perceived destination restorative qualities, well-being (hedonic experience and eudaimonic experience), destination image and loyalty. Through a questionnaire survey, 489 valid questionnaires were obtained and analyzed by structural equation model.

**Results:**

The results show that: (1) Perceived destination restorative qualities have a direct positive impact on hedonic experience, eudaimonic experience and destination image; (2) Both hedonic experience and eudaimonic experience have a direct positive impact on destination image and loyalty, in which the impact of hedonic experience is greater; (3) The relationship between destination image and loyalty is not significant.

**Discussion:**

The research results provide important guiding significance and enlightenment for the development of rural tourism.

## 1 Introduction

Nowadays, modern people are experiencing a great deal of stress and negative emotions as we face an accelerated pace of life, longer working hours and frequent global crises such as illness and economic recession (Kim et al., 2023). In this context, relieving psychological pressure and pursuing an active and healthy life has not only become a realistic demand of people, but also a subject worth deeper research (Lehto et al., 2017; Packer, 2021). In contrast to built-up cities, restorative destinations have beautiful natural landscapes, where individuals can escape from everyday life while rethinking their life goals, and maintain their working ability, increase cognitive flexibility, stimulate creativity and reduce fatigue (Bratman et al., 2015; Vujcic et al., 2019). Especially after the COVID-19 epidemic, the tourism demand of urban residents has gradually shifted from mass tourism to restorative tourism, and the demand for weekend travel vacations has been growing (Ding and Xu, 2024). Therefore, many urban planners and managers are developing and utilizing local natural and cultural resources to promote the development of restorative tourism products (He et al., 2021; Zhao and Li, 2023).

The perceived destination restorative qualities refer to the characteristics that tourists perceive in restorative tourism, which can improve their psychological, physical and social ability (Lehto, 2013). With the growth of research in restorative tourism, the research on the perceived destination restorative qualities is gradually shifting from the structural dimension to its consequences and influences (Backman et al., 2023; Li and He, 2023). Many studies have used Attention Restoration Theory (ART) to explain the influence of tourists’ perceived destination restorative qualities on their mental state and behavioral intention (Kim et al., 2014; Chen et al., 2024). Other researchers have chosen a different theoretical framework. For example, Kirillova and Wang (2016) apply the Social Presence Theory to explore whether the use of smartphones for social purposes (work and non-work related) during vacation enhances or hinders the potential of the tourist environment to promote a sense of resilience; Liu et al. (2024) adopted the Conservation of Resources Theory to study the complex relationship between the perceived destination restorative qualities of healthy resorts and the psychological recovery of tourists.

Although these research results provide significant insights for this study, there are still some shortcomings. Firstly, a majority of research focuses on artificial resorts, with relatively little discussion on rural tourism. Environmental psychology suggests that different types of environments trigger different perceived destination restorative qualities, which may influence subsequent effects at different levels (Zhang et al., 2024). Due to its rich natural and cultural landscape, rural tourism destinations can not only effectively improve the mood of tourists, but also stimulate their deep emotional resonance of the destination, so as to bring a deeper restorative experience (Chi and Han, 2021; Hong et al., 2024). Secondly, the measurement dimension of perceived destination restorative qualities mainly focuses on natural factors, and social factors are almost not included. Zhang et al. (2021) pointed out that in addition to natural factors, both social and symbolic landscapes have a significant impact on restorative experience. For example, a friendly destination can help visitors regain their attention by offering a variety of travel activities, while a cohesive community may have a better restorative effect (Chen et al., 2019; Feng et al., 2020). These findings suggest that restorative experiences stem from the interplay between physical and social factors (Scopelliti et al., 2016). Only by fully understanding the impact of all dimensions of perceived destination restorative qualities on tourists’ psychology and behavior can we carry out effective marketing and promote restorative tourism (Hu et al., 2022).

Previous research has developed multiple frameworks to explain the effects of perceived destination restorative qualities on tourists’ cognitive, emotional, and behavioral intentions in different tourism contexts (Cho et al., 2016; Chen and Xi, 2018). However, in terms of enhancing tourists’ hedonic experience, eudaimonic experience and destination image and promoting long-term loyalty, the role of perceived destination restorative qualities has not been fully studied. Based on this, this research focuses on the following three core issues. First, in the context of rural tourism, how do tourists’ perceived destination restoration quality affect their hedonic experience, eudaimonic experience and destination image? Secondly, what is the impact of tourists’ hedonic experience and eudaimonic experience on the destination image? Finally, what is the relationship between tourists’ hedonic experience, eudaimonic experience, destination image and loyalty? This study uses SOR paradigm to construct and verify this theoretical model, aiming at providing valuable contributions to the theoretical research and practical management of rural tourism.

## 2 Literature review and hypothesis development

### 2.1 SOR model

SOR model is an important framework for understanding the antecedents, interventions and outcomes of tourism activities in environmental psychology (Choi and Kandampully, 2019; Fakfare et al., 2024). According to the causal chain of SOR model, environmental stimuli affect the behavioral intention by affecting the cognitive and emotional states of the organism (Eroglu et al., 2001; Robert and John, 1982). Stimuli include internal and external environmental cues (Peng and Kim, 2014; Luqman et al., 2017), while organism refers to an individual’s cognitive and emotional states (such as pleasure, arousal and dominance), which mediate between stimuli and responses (Lin et al., 2023). The response was manifested as either positive approach behavior or negative avoidance behavior (Arora et al., 2020).

SOR model is widely applied in individual behavior research in heritage tourism, nature tourism, festival tourism and other fields (Hsu et al., 2021; Huang and Bu, 2022; Yang et al., 2022). On this basis, this study also applies SOR model to explain how tourists’ perceived destination restorative qualities affect their cognitive, emotional and behavioral intentions under the background of rural tourism. When tourists perceive the environment as restorative, this perception acts as a psychological stimulus, triggering their cognitive and emotional responses (Li and He, 2023). Therefore, this study considers the perceived destination restorative qualities as a stimulus, while traveler’s wellbeing and destination image are viewed as organic factors. Wellbeing is a comprehensive emotional experience, reflecting the overall emotional state of tourists during tourism, including hedonic experience and eudaimonic experience, both of which play an intermediary role in tourists’ behavior choices under environmental stimulation (Lee, 2024). Destination image represents tourists’ overall cognition and evaluation of the culture, landscape and services of a place, which not only affects their emotional response, but also further influences their behavioral intention (Jin et al., 2015; Kim, 2018). Finally, reactions focus on loyalty because reactions are expressed both in behavior and changes in attitude (Suhartanto et al., 2020).

### 2.2 Perceived destination restorative qualities

ART provides a solid theoretical and empirical basis for the analysis of individual restorative perception. According to ART, people must focus their attention to avoid distractions when dealing with various challenges in life, a process that relies on directed attention mechanisms (Kaplan and Kaplan, 1989). However, prolonged, high-intensity use of directed attention can lead to fatigue, so people need to recover attention in a restorative environment (Huang et al., 2022). Based on ART, Hartig et al. (1997) proposed the perceptual restorative scale for the first time, evaluating the restorative quality of perceived destination through four dimensions: fascination, being away, extent and compatibility. This framework has been further improved and verified by many scholars, and the dimensions of restorative perception have been expanded in different contexts (Bagot, 2004; Felsten, 2009; Cole and Hall, 2010). Zhai et al. (2023) further proposed that restorative originates from the interaction between physical and social factors, adding the dimension of “life atmosphere” and expanding the original four-dimensional scale. Specifically, being away means withdrawing from an environment that causes fatigue and loss of concentration (Cho et al., 2016). Fascination is the ability to easily attract attention to an environment (Scopelliti and Vittoria Giuliani, 2004). Compatibility means that the environment fits with personal preferences and brings a sense of belonging (Herzog et al., 2003). Extent is related to the richness of environmental resources (Cole and Hall, 2010). And Life atmosphere captures the mood and spiritual character derived from local production and living conditions. It is defined by two key components: friendliness, which reflects tourists’ subjective impressions of the warmth and approachability in local interpersonal interactions, and vitality, which denotes the direct perception of local residents’ lifestyles and conditions. The interplay of these elements fosters a sense of social relaxation, thereby promoting restoration (Zhai et al., 2023). This five-dimension scale provides a powerful measurement tool for assessing the restorative perception of the rural tourism environment.

### 2.3 Perceived destination restorative qualities and wellbeing

Wellbeing is a comprehensive concept that covers the subjective evaluation of overall life satisfaction, satisfaction in various areas, emotional and social wellbeing, and the evaluation of psychological resources (Huang et al., 2024). According to different philosophical origins, wellbeing can be divided into hedonic experience and eudaimonic experience (Lee, 2024). Hedonic experience mainly focuses on the emotional response or pleasure brought by tourism products, which usually reflects short-term emotional benefits (Wang et al., 2020). In contrast, eudaimonic experiences are more focused on mental health and quality of life enhancement, and are often related to self-eudaimonic, human development, personal purpose, and virtue (Sirgy and Uysal, 2016). Lengieza et al. (2019) pointed out that in current tourism research, hedonic experience is mainly reflected in two dimensions of pleasure and avoidance, while eudaimonic experience includes two dimensions of personal meaning and self-reflection.

Highly restorative environments, such as natural landscapes, quiet parks or comfortable leisure areas, can effectively relieve individual fatigue and stress and promote emotional recovery (Chen et al., 2024). Baldwin et al. (2021) pointed out that individuals exposed to the natural environment are more likely to experience emotional recovery and enhancement of positive emotions, thus enhancing their hedonic experience. In the context of developing countries, Zhang et al. (2024) found that in suburban national forest parks, there was a positive correlation between perceived restorativeness and general subjective wellbeing (i.e., hedonic experience).

According to ART, natural and restorative environments can help restore focus, reduce mental fatigue, and promote deep thinking (Hartig et al., 2003; Kaplan and Kaplan, 1989). These environments provide a space for individuals to relax, think and reflect, helping to re-examine their own goals and meaning in life, thereby enhancing their sense of wellbeing (Huang et al., 2022). Furthermore, Løvoll et al. (2020) point out that there is a significant positive correlation between tourists’ positive perception of the destination environment, such as beauty, comfort and naturalness, and their hedonic and eudaimonic feelings. In summary, this paper proposes the following hypotheses:

*H1*. Perceived destination restorative qualities have a significant positive effect on hedonic experience.

*H2*. Perceived destination restorative qualities have a significant positive impact on eudaimonic experience.

### 2.4 Perceived destination restorative qualities and destination image

Destination image is usually defined as a tourist’s psychological perception of the knowledge, attributes and overall impression of a tourist attraction (Fakeye and Crompton, 1991). Chi and Han (2021) pointed out that a highly restorative environment can not only improve tourists’ mood and psychological state, but also enhance their favorable impression of the destination. These positive emotional experiences are crucial to shaping a positive image of the destination (Joo et al., 2023). At the same time, Prayag et al. (2017) analyzed the relationship between emotional experience and destination image, and proposed that restorative environment can improve tourists’ overall destination image. Based on the above research, this study proposes the following hypothesis:

*H3*. Perceived destination restorative qualities have a significant positive impact on destination image.

### 2.5 Wellbeing and destination image

In tourism research and development, the idea of improving tourist wellbeing does not conflict with the goal of improving destination image (Aureliano-Silva et al., 2017). The pleasure and emotional satisfaction obtained by tourists during the travel process will improve their overall evaluation and cognition of the destination (Chi et al., 2022). This positive emotional experience reinforces the image of the destination in the minds of tourists, increases their willingness to revisit, and influences the perception of others about the destination through word of mouth (Joo et al., 2023). As Kim and Fesenmaier (2015) pointed out, the happiness and satisfaction experienced in tourism directly affect tourists’ cognition and attitude toward the destination image.

Visitors tend to have a deeper emotional connection with the destination when they gain a sense of personal growth and meaning (Ali et al., 2014). This deep experience not only deepens the tourists’ positive evaluation of the destination, but also further enhances the overall awareness of the destination’s image (Liu and Huang, 2020). Pung and Chiappa (2020) pointed out that the transformational travel experience promotes tourists’ eudaimonic experience, thus enhancing the perceived image of the destination. Similarly, Kuykendall et al. (2015) explored the role of self-actualization and psychological satisfaction in the travel experience, finding that these factors have a significant positive impact on the image of a destination, especially in the context of deep cultural and natural experiences. Therefore, this paper proposes the following hypotheses:

*H4.* Hedonic experience has a significant positive impact on destination image.

*H5.* Eudaimonic experience has a significant positive impact on destination image.

### 2.6 Wellbeing and loyalty

Loyalty is defined as an individual’s willingness and behavior to continue to use, consume, or patronize a product, service, or destination in the future (Oliver, 1999). In the process of travel, if tourists obtain higher subjective wellbeing, their sense of pleasure will increase, thus forming a higher loyalty (Wang et al., 2020). Balleyer and Fennis (2022) showed that hedonism is the key factor affecting people’s purchase of tourism and leisure products. At the same time, Lee (2024) pointed out that eudaimonic experience can help tourists better understand themselves, the world and their position in the world, so as to enhance their willingness to revisit and recommend the place. Further research also supported this view, and Al-okaily et al. (2023) found that achieving wellbeing has a direct impact on tourists’ destination loyalty. Keskin et al. (2024) showed that in the context of food festivals, high levels of hedonic experience and eudaimonic experience can enhance the influence of loyalty on behavioral intention. In summary, this paper proposes the following hypotheses:

*H6.* Hedonic experience has a significant positive effect on loyalty.

*H7.* Eudaimonic experience has a significant positive impact on loyalty.

### 2.7 Destination image and loyalty

A sound destination image can attract new visitors, and also cultivate loyal customers and destination promoters, thereby strengthening its position in a competitive tourism market (Benítez-Márquez et al., 2021; Chi et al., 2022). Although the positive correlation between destination image and tourist loyalty has been verified many times, the research on this relationship in the context of rural tourism is still limited (Zhang et al., 2014; Jeong and Kim, 2019). Therefore, this study proposes the following hypothesis:

*H8.* Destination image has a significant positive impact on loyalty.

**Figure 1:**
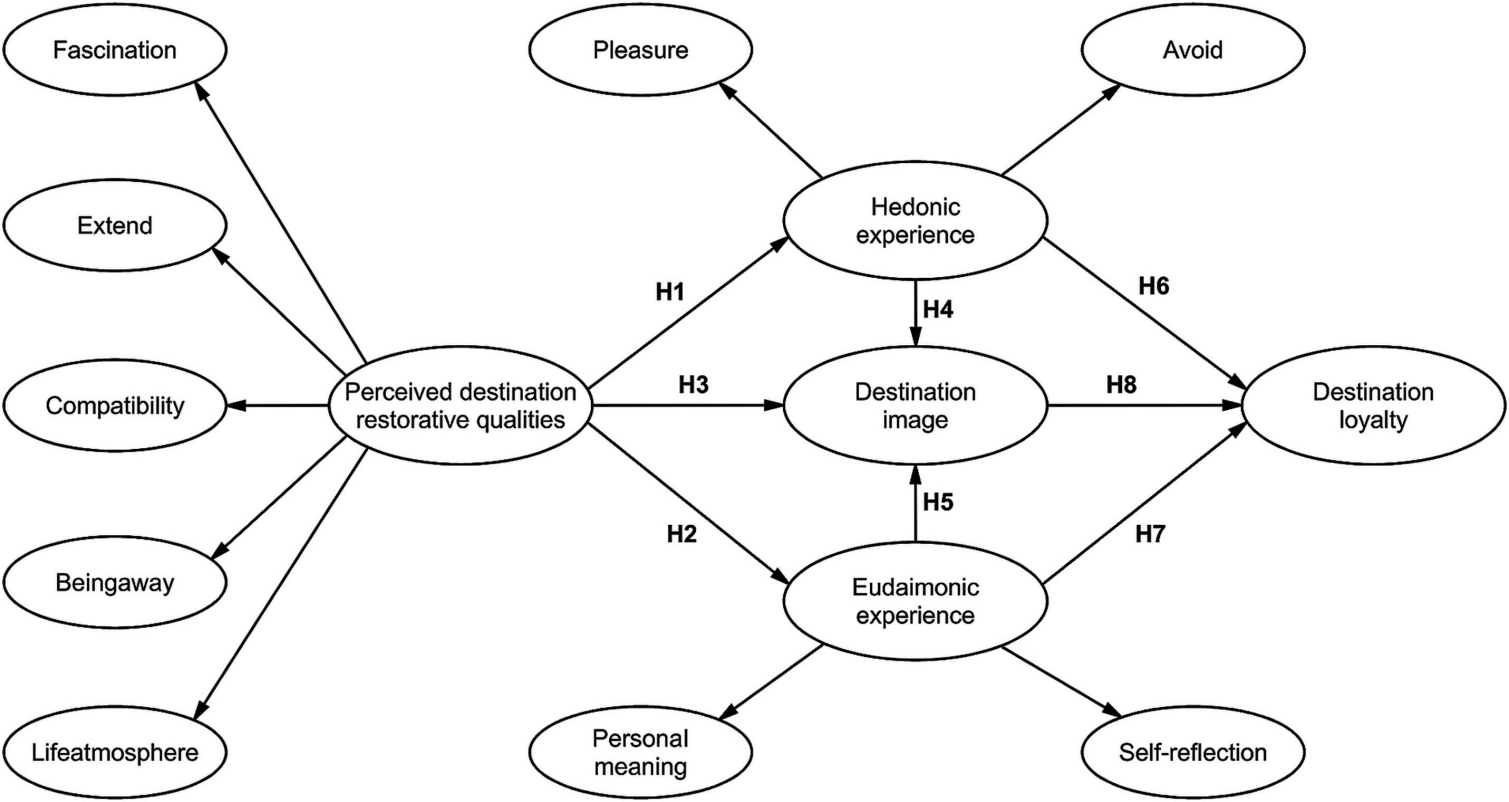
Conceptual model.

Grounded on the above research and assumptions, a conceptual model is presented in Figure 1.

## 3 Methods

### 3.1 Study site and target subjects

Located in Huangdao District, Qingdao City, Shandong Province, Yumingzui village is a traditional fishing village with a history of more than 300 years, surrounded by the sea on three sides and the mountains on one side. Local residents make full use of the geographical advantage and live a life combining agriculture and fishing. Although the village has been renovated in recent years and the whole village residents have moved to a nearby town, the villagers are still engaged in fishing activities here and sell seafood in the scenic spot, attracting a large number of tourists to come and buy. In addition, real estate has been developed on the original site, and villa groups have been built to form a rural tourism resort integrating business, catering and accommodation, providing tourists with an ideal residence for leisure and slow life. The coastal reefs provide an ideal spot for anglers, while the seaside lawns attract a large number of camping tourists.

The subjects of this study are tourists who went to Yumingzui village for vacation in 2024. The researchers used convenience sampling to collect data from voluntarily participating visitors at resort areas. Each visitor took about 15 to 20 min to complete the questionnaire. In addition, both the pilot survey and the primary data collection were aimed at visitors over the age of 18. Finally, a total of 489 valid samples were collected, which exceeded the minimum sample size recommended by Weston and Gore (2006).

### 3.2 Scales and measuring tools

The researchers of this study compiled a structured questionnaire by reviewing the relevant literature. The questionnaire consisted of six parts: (1) perceived destination restorative qualities, (2) hedonic experience, (3) eudaimonic experience, (4) destination image, (5) loyalty, and (6) background information of the interviewees. The 17 items of the perceived destination restorative qualities scale were verified in previous studies and modified appropriately according to the specific situation of Yumingzui village (Zhai et al., 2023). The 12-item scale created by Lengieza et al. (2019) was used to measure eudaimonic experience and hedonic experience. To assess tourists’ perception of a destination’s image, four items from a previous study were adapted (Chi and Han, 2021; Davras and Özperçin, 2023). The measurement of loyalty was based on four items in the previous research on rural tourism loyalty (Chen et al., 2024). The sixth part collected personal information such as gender, age and education level of visitors. All items were assessed using a five-point Likert scale, ranging from “strongly disagree” (=1) to “strongly agree” (=5).

Two Master’s students proficient in English independently translated the original English scale into Simplified Chinese (Mandarin). Subsequently, the research team compared the two translations, discussed and revised the discrepancies, and ultimately produced a unified Chinese version. Thereafter, two Master’s students fluent in both Chinese and English, who had not been exposed to the original English scale, conducted a back-translation of the Chinese version. The research team evaluated the back-translation results and convened an expert review committee for assessment. This expert review committee comprised both translation specialists and experts in the field of tourism. Prior to formal data collection, a pre-test was conducted to guarantee the reliability and validity of the questionnaire. Both the pre-test participants and the formal survey subjects were tourists on vacation in Yumingzui Village, who were invited to participate in the pre-test after consent. Data collection was conducted from April 6 to April 15, 2024, using the convenience sampling method. The target respondents were 120 people, and 103 valid questionnaires were eventually collected, with a response rate of about 86%. Subsequently, the data were evaluated for reliability. The results showed that Cronbach’s alpha reliability coefficient of each scale ranged from 0.73 to 0.91, indicating that the questionnaire had good reliability. As a result, all 37 items were retained in the official data collection.

### 3.3 Data collection and analysis

The official data collection was conducted in Yumingzui village from April 27 to May 13, 2024, and the research team selected a total of 550 tourists to participate in the questionnaire. To ensure the availability of reliable samples, the researchers carried out an in-depth search for missing values and outliers. During the data preprocessing stage, a stringent filtering mechanism was implemented for the questionnaires. Specifically, any response with a time shorter than 180 s, as well as cases containing outliers, were systematically removed. Through this meticulous data-cleaning process, a total of 489 valid cases were retained, forming a solid basis for subsequent in-depth analysis. According to the data analysis, there were slightly more female respondents than men, accounting for 51.33 and 48.67%, respectively. The majority of respondents had a university degree (75.46%), of which 6.54% had a postgraduate degree. In terms of income, 74.48 percent of respondents earned less than 8,000 yuan a month, and 8.38 percent earned more than 15,000 yuan. The age of tourists is concentrated between 18 and 60 years old. The occupational distribution is more diverse. Table 1 details the demographic characteristics of the respondents.

**Table 1:** Research participants’ general characteristics.

Variable	Category	Number	%
Gender	Female	251	51.33
	Male	238	48.67
Age	18–30	217	44.38
	31–40	142	29.04
	41–60	113	23.11
	Over 60	17	3.47
Educational background	Junior high school or below	27	5.52
	High school or technical secondary school	93	19.02
	College or undergraduate	337	68.92
	Graduate	32	6.54
Income(CNY)	Below 2000	46	9.04
	2001–5,000	113	23.11
	5,001–8,000	207	42.33
	8,001–15,000	82	16.8
	Over 15,000	41	8.38
Profession	Enterprise workers	261	53.37
	Civil servants, public institution workers	59	12.07
	Self-employed	71	14.52
	Student	44	9.00
	Retiree	38	7.77
	Other	16	3.27

1 CNY equals approximately 0.14 USD.

Data analysis was performed using SPSS 27.0 and AMOS 26.0. The test of the measurement model is divided into two steps. First, the reliability and validity of the CFA tool are tested. Secondly, Cronbach’s alpha coefficient was calculated to check the internal consistency of each subscale. The research model was verified by structural equation modeling (SEM), and finally the relationship between the direct effects, indirect effects and total effects was calculated. The efficacy and strength of our SEM were meticulously evaluated using a range of well-established fit indices, including the Chi-Square to Degrees of Freedom Ratio, the Tucker-Lewis Index (TLI), the Comparative Fit Index (CFI), the Standardized Root Mean Square Residual (SRMR), and the Root Mean Square Error (RMSE) of Approximation (Schermelleh-Engel et al., 2003). A model was considered adequate when the TLI and the CFI reached or exceeded 0.95, and SRMR as well as the RMSE of Approximation remained below 0.05, thereby affirming the model’s validity and reliability (Schermelleh-Engel et al., 2003).

## 4 Results

The multivariate normal distribution was tested as a basic assumption when conducting structural equation modeling. According to Kline (2015), a normal distribution is assumed when the absolute value of the skewness index is less than 3, and the absolute value of the kurtosis is less than 10. The values for skewness ranged between 0.036 and 1.176, while the kurtosis ranged between 0.035 and 1.268, suggesting that the data in this study are normally distributed. In addition, linear regression was used to detect multicollinearity between variables. By calculating the tolerance values and variance inflation factors (VIF), all VIF values were less than 10 and all tolerance values were greater than 0.1, indicating that there is no potential problem of multicollinearity among the independent variables in this study (Thompson et al., 2017).

### 4.1 Measurement model

The measurement model’s reliability and validity was assessed by the metrics below: standardized factor loadings, Cronbach’s alpha coefficient, convergent validity, discriminant validity, as well as model fit.

From the study by Schumacker and Lomax (2004), the standardized factor loadings of the items should not be below 0.50. Though the standardized factor loadings of the whole items in this study were higher than 0.50 (ranging from 0.516 to 0.930), LA1 and DI4 were deleted to improve the fit of the model. The Cronbach’s alpha coefficients for all five constructs exceeded 0.70 (0.748 to 0.912) (Table 2), showing a good internal consistency.

**Table 2:** Analysis results of construct validity and reliability.

Constructs/Variables		Mean	Std.Dev	Factor Loadings	α	CR	AVE
Perceived destination restorative qualities							
Fascination		3.06	0.74		0.858	0.858	0.668
FA1	There are a lot of interesting things that catch my attention.			0.830			
FA2	I think the view is fascinating.			0.806			
FA3	I do not feel bored here.			0.815			
Extent		3.06	0.76		0.848	0.849	0.651
EX1	I think the scenery here is in harmony with the environment.			0.791			
EX2	I think people live in harmony with nature.			0.809			
EX3	I feel like there is a lot to do here.			0.821			
Being away		3.03	0.77		0.846	0.847	0.649
BA1	I am far away from my daily life here.			0.817			
BA2	I am away from the stresses of life.			0.841			
BA3	I focus on the trip and do not have to worry about other things.			0.757			
Compatibility		3.34	0.67		0.777	0.775	0.535
CO1	I feel a sense of belonging here.			0.713			
CO2	What I’ve done here is what I want to do.			0.720			
CO3	I handle the trip with ease.			0.761			
Life atmosphere		3.18	0.76		0.907	0.895	0.648
LA2	I feel that the environment is quiet and peaceful.			0.895			
LA3	I feel very warm interacting with the residents.			0.690			
LA4	I feel like the life pace here is slow.			0.770			
LA5	I feel that the lifestyle is very simple.			0.930			
Eudaimonic experience							
Personal meaning		3.40	0.70		0.808	0.808	0.583
PM1	This trip helps me think about my true potential.			0.783			
PM2	This trip helps me grow as a person.			0.747			
PM3	This trip gives me a sense of purpose in my life.			0.761			
Self-reflection		3.44	0.67		0.774	0.777	0.540
SR1	I have time for self-reflection.			0.761			
SR2	I think about the meaning of life on this trip.			0.795			
SR3	I can think deeply about topics I care about.			0.639			
Hedonic experience							
Pleasure		3.45	0.62		0.748	0.752	0.505
PL1	I have many laughs on this trip.			0.612			
PL2	This trip is entertaining.			0.735			
PL3	This trip makes me happy.			0.774			
Avoid		3.05	0.72		0.807	0.808	0.584
AV1	This trip helps me forget the problems in the world.			0.785			
AV2	This trip helps me get away from negative news in the papers, TV, internet postings, etc.			0.737			
AV3	This trip allows me to live like I do not have a care in the world.			0.771			
Destination image		3.07	0.80		0.845	0.850	0.655
DI1	The overall image I have of this tourism destination is favorable.			0.798			
DI2	My overall image of this tourism destination is positive.			0.731			
DI3	There are beautiful natural landscapes.			0.891			
Destination loyalty		3.27	0.73		0.912	0.913	0.725
DL1	I will revisit this tourist destination in the future.			0.897			
DL2	I will probably revisit this tourist destination in 2 years.			0.786			
DL3	I will recommend this tourist destination to friends and relatives.			0.846			
DL4	I will say positive things about this tourist destination to others.			0.873			

FA: fascination, EX: extent, CO: compatibility, BA: being away, LA: life atmosphere, PM: personal meaning, SR: Self-reflection, PL: Pleasure, AV: avoid, DI: destination image, DL: destination loyalty.

From the research by Fornell and Larcker (1981), for all the constructs, the CR should not be below 0.70 and the AVE should exceed 0.50, and the five constructs in this research met the two criteria (Table 3). In terms of the discriminant validity of the constructs, the square root of the AVE must exceed the correlation coefficient between the construct and the other constructs, and this criterion was met by the present measurement model, implying its good discriminant validity (Table 3).

**Table 3:** Discriminate validity of the research model.

	FA	EX	CO	BA	LA	PM	SR	PL	AV	DI	DL
FA	0.817										
EX	0.448	0.807									
CO	0.389	0.344	0.731								
BA	0.408	0.38	0.404	0.806							
LA	0.324	0.318	0.383	0.249	0.805						
PM	0.087	0.097	0.121	0.019	0.046	0.764					
SR	0.043	0.121	0.118	0.007	0.066	0.444	0.735				
PL	0.186	0.188	0.251	0.197	0.223	0.243	0.203	0.711			
AV	0.171	0.163	0.241	0.198	0.211	0.065	0.063	0.377	0.764		
DI	0.353	0.356	0.305	0.270	0.314	0.181	0.177	0.345	0.202	0.809	
DL	0.162	0.147	0.135	0.098	0.166	0.203	0.185	0.260	0.159	0.232	0.851

FA: fascination, EX: extent, CO: compatibility, BA: being away, LA: life atmosphere, PM: personal meaning, SR: self-reflection, PL: pleasure, AV: avoid, DI: destination image, DL: destination loyalty.

Combined with the criteria recommended by Hu and Bentler (1999), in this research, the fit of the measurement model was satisfactory, χ^2^/df = 1.347, TLI = 0.976, CFI = 0.979, RMR = 0.025, RMSEA = 0.027 (Table 4).

**Table 4:** The goodness of fit indices for the measurement model and research model.

Model	x^2^	x^2^/df	TLI	CFI	RMR	RMSEA
Measurement model	678.883 (0.000)	1.347	0.976	979	0.025	0.027
Research model	748.439 (0.000)	1.381	0.973	0.976	0.032	0.028
Recommended criteria	*p*>0.05	<5.0	>0.90	>0.90	<0.05	<0.08

### 4.2 Structural model

SEM validation of the research model was conducted. The research results showed that the model’s fit was χ^2^/df = 1.381, TLI = 0.973, CFI = 0.976, RMR = 0.032, and RMSEA = 0.028 (Table 4). The test results showed that all seven hypotheses were verified except H7 (Table 5).

**Table 5:** The hypothesis test results.

Hypothesis	Hypothesized path	B	β	S.E	t	Result
H1	PD → EE	0.212	0.213	0.071	3.006**	Supported
H2	PD → DI	0.637	0.417	0.115	5.549***	Supported
H3	PD → HE	0.524	0.521	0.079	6.641***	Supported
H4	EE → DI	0.236	0.154	0.092	2.577*	Supported
H5	HE→DI	0.320	0.211	0.118	2.703**	Supported
H6	EE → DL	0.329	0.230	0.096	3.419***	Supported
H7	DI → DL	0.081	0.087	0.058	1.396	Rejected
H8	HE→DL	0.370	0.262	0.108	3.432***	Supported

PD: perceived destination restorative qualities, EE: eudaimonic experience, HE: hedonic experience, DI: destination image, DL: destination loyalty. ***p < 0.001, **p < 0.01, *p < 0.05.

**Figure 2:**
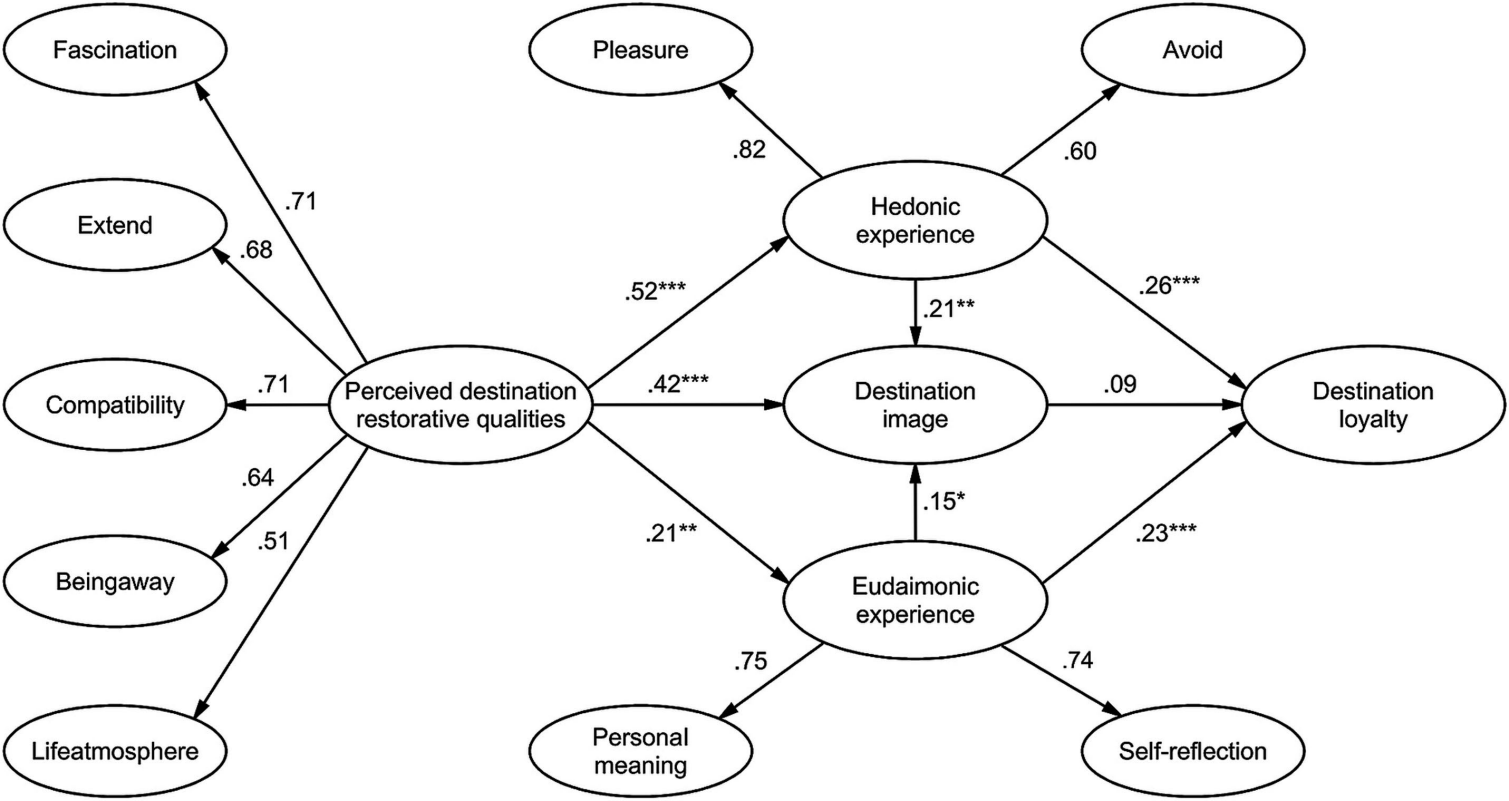
Structural model. *** Significant at *p* < 0.001, ** significant at *p* < 0.01, * significant at *p* < 0.05.

Perceived destination restorative qualities were significantly and positively correlated with eudaimonic experience (*β* = 0.213, *t* = 3.006, *p* < 0.01), destination image (*β* = 0.417, *t* = 5.549, *p* < 0.001), and hedonic experience (*β* = 0.678, *t* = 6.641, *p* < 0.001), which supported Hypotheses H1, H2, and H3. Eudaimonic experience was also significantly and positively correlated with destination image (*β* = 0.154, *t* = 2.577, *p* < 0.05) and tourist loyalty (*β* = 0.230, *t* = 3.419, *p* < 0.001), supporting hypotheses H4 and H6. Hedonic experience was also positively correlated with destination image (*β* = 0.211, *t* = 2.703, *p* < 0.01) and tourist loyalty (*β* = 0.262, *t* = 3.432, *p* < 0.001), supporting hypotheses H5 and H8. However, destination image was not significantly associated with tourist loyalty (*β* = 0.087, *t* = 1.396, *p* > 0.05), negating hypothesis H7. The validated structural model is shown in Figure 2.

### 4.3 Direct and indirect aggregation impacts between variables

The indirect, direct, and overall impacts between perceived destination restorative qualities, eudaimonic experience, destination image, hedonic experience, and destination loyalty were recalculated after excluding non-significant correlation (Table 6). The results showed that perceived destination restorative qualities had a weight of 0.234 on destination loyalty mediated by eudaimonic experience and hedonic experience, with hedonic experience having the largest indirect effect (*β* = 0.521 × 0.262 = 0.137), eudaimonic experience having the second largest indirect effect (*β* = 0.154 × 0.230 = 0.035), and destination image having the smallest indirect effect (*β* = 0.211 × 0.087 = 0.018).

**Table 6:** Direct, indirect, and total effects among the variables.

Dependent variable	Independent variable	Direct effect	Indirect effect	Total effect	*R* ^2^
FA	PD	0.708***	–	0.708	0.501
EX	PD	0.679***	–	0.679	0.461
CO	PD	0.712***	–	0.712	0.507
BA	PD	0.644***	–	0.644	0.415
LA	PD	0.511***	–	0.511	0.261
PM	PD	–	0.160	0.160	0.565
	EE	0.752***	–	0.752	
SR	PD	–	0.157	0.157	0.545
	EE	0.738***	–	0.738	
PL	PD	–	0.426	0.426	0.668
	HE	0.818***	–	0.818	
AV	PD	–	0.313	0.313	0.361
	HE	0.601***	–	0.601	
EE	PD	0.213**	–	0.213	0.045
HE	PD	0.521***	–	0.521	0.272
DI	PD	0.417***	0.143	0.560	0.368
	EE	0.154*	–	0.154	
	HE	0.211**	–	0.211	
DL	PD	–	0.234	0.234	0.173
	EE	0.230***	0.013	0.243	
	DI	0.087	–	0.087	
	HE	0.262***	0.018	0.280	

FA: fascination, EX: extent, CO: compatibility, BA: being away, LA: life atmosphere, PD: perceived destination restorative qualities, PM: personal meaning, SR: Self-reflection, PL: Pleasure, AV: avoid, EE: eudaimonic experience, HE: hedonic experience, DI: destination image, DL: destination loyalty. ***p < 0.001, **p < 0.01, *p < 0.05.

## 5 Discussion and conclusion

### 5.1 Research conclusions

Based on SOR model, this study explores the impact of perceived destination restorative qualities on tourists’ hedonic experience, eudaimonic experience, destination image and loyalty, and verifies these relationships through empirical research. The results are as follows:

The perceived destination restorative qualities of rural tourism destinations have significant positive effects on tourists’ hedonic experience, eudaimonic experience and destination image. According to ART, the natural environment can effectively relieve the mental fatigue of tourists and improve their concentration and mental resources (Lee et al., 2015). Therefore, the sense of relaxation and pleasure in the rural environment significantly enhances the pleasure-seeking experience of visitors. Rural tourism provides an environment for tourists to relax, and more importantly, it creates a space for self-reflection (Sang, 2020). In the quiet and close-to-nature environment, it is easier for visitors to engage in inner thinking and self-examination, thereby reassessing life goals, values and their own potential, and enhancing the realization experience (Løvoll et al., 2020; Ding and Xu, 2024). Besides, rural tourism also incorporates rich cultural and social interactions, such as village folk activities and the warm reception of local residents (Chi and Han, 2021). These cultural and interpersonal experiences further deepen tourists’ goodwill and emotional connection with the destination and shape a more positive image of the destination.

Hedonic experience has a significant positive impact on destination image and loyalty, indicating that a pleasant and relaxed experience is the key factor to enhance tourist identity and loyalty. In contrast, although eudaimonic experience also has a positive impact on destination image and loyalty, its direct impact is weak, which may be due to that the immediacy of hedonic experience and strong emotional experience are more likely to directly affect tourists (Lee, 2024). Existing studies mainly focus on hedonistic wellbeing, with relatively few studies on self-achievement and personal development (Wang et al., 2020; Zhang et al., 2024). This study, for the first time, comprehensively examines the experience of pleasure and eudaimonic in rural tourism, and the results show that the hedonic experience strengthens the emotional connection between tourists and the destination, while the eudaimonic experience enhances the sense of identity of tourists through personal growth.

Although many tourism studies regard destination image as an important factor affecting loyalty (Zhang et al., 2014; Chi and Han, 2021), this study finds that its direct impact on loyalty is not significant, which may be related to the research background, sample selection, cultural differences, and differences in loyalty measurement methods (Charinsarn et al., 2023; Qi et al., 2023). In rural tourism, research shows that tourists often prioritize personal restorative experiences and emotional bonds over a straightforward cognitive evaluation of destination image (Huang and Bu, 2022; Ding and Xu, 2024). Moreover, studies by Chiu et al. (2016) and Tasci et al. (2022) indicate that factors like emotional attachment, satisfaction, and place attachment can moderate this relationship. These studies provide a theoretical basis for interpreting our results and suggest that the impact of destination image on loyalty may not always be direct.

### 5.2 Research contributions

By incorporating social factors such as cultural interaction and community cohesion, we address a dimension of restorative tourism that has traditionally centered on natural or physical environments, thereby offering a more comprehensive understanding of the rural tourism experience. The application of the SOR model in a rural setting further clarifies how environmental stimuli affect visitors’ psychological responses, extending the scope of environmental psychology in tourism research. We also investigate the mediating roles of hedonic and eudaimonic wellbeing between perceived restorative qualities and loyalty, addressing a gap in the literature that often focuses on a single dimension of wellbeing. Moreover, the finding that destination image does not directly influence loyalty challenges prevailing assumptions and suggests that emotional factors and overall experience quality may weigh more heavily than cognitive evaluations of destination image, particularly in rural tourism contexts.

### 5.3 Management implications

This study confirms a positive relationship between the five dimensions of perceived environmental restorativeness and tourist loyalty, highlighting its crucial role in rural tourism development. Destinations can enhance tourist relaxation, interest, and exploration by creating tranquil nature areas, incorporating multisensory designs, and offering diverse activities for different visitor groups (e.g., families, backpackers, senior travelers). Additionally, developing ecotourism, hiking, fruit picking, and camping can further enrich tourist experiences and satisfaction. Moreover, the study reveals that wellbeing (both hedonic and eudaimonic) plays a key mediating role between perceived environmental restorativeness and loyalty. Therefore, rural tourism destinations should balance relaxation and personal growth by designing integrated experiences that combine cultural activities with nature-based healing. This approach enables tourists to achieve both enjoyment and self-fulfillment, ultimately enhancing their overall wellbeing and loyalty.

## 6 Limitations and future research direction

There are some limitations to our findings. First of all, this study employed convenience sampling and the sample was primarily drawn from a single rural tourism destination in China, the representativeness of the sample is relatively limited, thereby constraining the external validity of the findings. Future research should consider using more representative sampling methods, such as stratified sampling or multi-site random sampling, and expand the scope to include other rural tourism destinations, as well as settings with different cultural backgrounds or urban tourism environments, to enhance the reliability and generalizability of the results.

Secondly, this study focuses exclusively on short-term tourism experiences and does not consider the potential influence of long-term tourism experiences on tourist loyalty and restorative outcomes. The experiences and behaviors of long-term tourists may differ significantly from those of short-term tourists. Consequently, future research should differentiate between these two groups and examine the distinct effects of long-term versus short-term tourism experiences on tourist loyalty.

Thirdly, this study did not consider factors such as seasonal effects, marketing influence, or returning customer bias, all of which may significantly affect tourists’ perceptions of restorative quality and loyalty. Seasonal variations in tourism activities, destination marketing strategies, or the unique experiences of repeat visitors may influence tourists’ loyalty (Almeida-Santana and Moreno-Gil, 2017; Meleddu et al., 2015; Jin et al., 2015). Future research should incorporate these factors by employing longitudinal or cross-seasonal data collection methods to further investigate the impact of these external variables on tourists’ loyalty. Finally, our study did not consider other psychological constructs like place attachment and emotional bonding that could influence loyalty. Future research should examine how these factors mediate the link between perceived environmental restoration and loyalty.

## Data Availability

The original contributions presented in the study are included in the article/Supplementary material, further inquiries can be directed to the corresponding author/s.
